# *MicroRNA-708-3p* as a potential therapeutic target via the *ADAM17-GATA/STAT3* axis in idiopathic pulmonary fibrosis

**DOI:** 10.1038/emm.2017.311

**Published:** 2018-03-30

**Authors:** Bo Liu, Rongrong Li, Jinjin Zhang, Chao Meng, Jie Zhang, Xiaodong Song, Changjun Lv

**Affiliations:** 1 Department of Respiratory Medicine, Affiliated Hospital to Binzhou Medical University, Binzhou, China; 2 Department of Cellular and Genetic Medicine, School of Pharmaceutical Sciences, Binzhou Medical University, Yantai, China

## Abstract

MicroRNAs (miRNAs) are important diagnostic markers and therapeutic targets for many diseases. However, the miRNAs that control the pathogenesis of idiopathic pulmonary fibrosis (IPF) and act as potential therapeutic targets for the disease are rarely studied. In the present study, we analyzed the function and regulatory mechanism of *microRNA-708-3p* (*miR-708-3p*) and evaluated this marker’s potential as a therapeutic target in IPF. The clinical and biological relevance of fibrogenesis for *miR-708-3p* was assessed *in vivo* and *in vitro*, specifically in matching plasma and tissue samples from 78 patients with IPF. The data showed that the *miR-708-3p* levels decreased during fibrosis and inversely correlated with IPF. The experiments showed that the decreased *miR-708* promoter activity and *primer-miR-708*(*pri-miR-708*) expression were the potential causes. By computational analysis, a dual luciferase reporter system, rescue experiments and a Cignal Finder 45-Pathway system with *siADAM17* and a *miR-708-3p* mimic, we identified that *miR-708-3p* directly regulates its target gene, a disintegrin and metalloproteinase 17 (*ADAM17*), through a binding site in the 3′ untranslated region, which depends on the *GATA/STAT3* signaling pathway. Finally, an *miR-708-3p* agomir was designed and used to test the therapeutic effects of the *miR-708-3p* in an animal model. Small-animal imaging technology and other experiments showed that the dynamic image distribution of the *miR-708-3p* agomir was mainly concentrated in the lungs and could block fibrogenesis. In conclusion, the *miR-708-3p*–*ADAM17* axis aggravates IPF, and *miR-708-3p* can serve as a potential therapeutic target for IPF.

## Introduction

Idiopathic pulmonary fibrosis (IPF) is a specific form of chronic, progressive fibrosing interstitial pneumonia in adults.^1^ The America Heart, Lung, and Blood Institute recommends strategic planning and future research to improve the definition of IPF pathogenesis and develop effective therapies.^2^ One recommendation is identifying how microRNA (miRNA) and pathway changes result in IPF. MiRNAs are a large family of regulatory RNAs that inhibit target gene expression by base pairing with complementary sites in the 3′ untranslated regions (UTR) to promote mRNA decay and translational repression.^3^ MiRNA is critical in the development and dysregulation that leads to diseases, as well as being an important diagnostic marker and therapeutic target.^4, 5^

The first report on abnormal miRNA in cancer involved *miRNA-143* and *miRNA-145* downregulation in colorectal neoplasia.^6^ Similar findings were reported for pancreatic cancer, breast cancer and other solid tumors of epithelial origin.^7, 8, 9^ In cancer pathogenesis, the significant role of miRNAs was uncovered by examining human tumor samples. Virtually all of the examined tumor types were characterized by globally abnormal miRNA expression patterns, and miRNA expression profiles provided significant information on tumor classification, prognosis and therapeutic response.^10, 11, 12^ IPF exhibits several cancer-like pathogenic features and is a neoproliferative lung disorder. Fundamental pathogenic hallmarks for both IPF and cancer include epigenetic and genetic abnormalities and functional features, such as uncontrolled proliferation, resistance to apoptosis and high migration rates.^13, 14, 15^ However, unlike other studies on miRNAs in cancers, studies have not revealed the precise miRNA regulation and mechanism controlling IPF pathogenesis.

*MicroRNA-708-3p* (*miR-708-3p*) is highly conserved across species, but its role and mechanism in IPF have not been established to date. This paper follows up on our previous laboratory research.^16^ In the present study, we focused on the therapeutic target and mechanism of *miR-708-3p* and analyzed its target gene and signaling pathway during IPF development. Along with clinical data from patients with IPF, this study provided a rationale for developing *miR-708-3p* as a therapeutic target for IPF.

## Materials and methods

### Patients and healthy volunteers

IPF is diagnosed in accordance with the American Thoracic Society/European Respiratory Society consensus criteria,^1^ which include clinical, radiographic and characteristic histopathological features (*n*=78). A blood sample (5 ml) was obtained from each participant and prepared for testing. Matching plasma samples from healthy volunteers (*n*=78) were then selected on the basis of patient sex and age. Written informed consent was obtained by doctors from each participant. This study was approved by the ethics committee of Binzhou Medical University.

### Animal model and ethics statement

Mice with a mean weight of 25±2 g were purchased from the Model Animal Research Center of Nanjing University (Nanjing, China). All animal experiments were performed under the regulations of the Committee on the Ethics of Animal Experiments of Binzhou Medical University.^17^ The mice were housed under a 12 h light/dark cycle, allowed free access to food and water and randomly divided into four groups (10 mice per group): sham group, bleomycin-treated group (BLM group), BLM+ negative control for the *miR-708-3p* agomir group (NC group), and BLM+ *miR-708-3p* agomir group. Each mouse was administered 10 nmol *miR-708-3p* agomir, which was sprayed into the lungs nine times at intervals of 2 days using a Penn-Century MicroSprayer (Penn-Century Inc., Wyndmoor, PA, USA). At day 28, all mice were killed, and lung tissue sections were collected and immediately frozen in liquid nitrogen for further studies. The BLM animal model was administered 5 mg kg^−1^ BLM dissolved in saline via a single intratracheal instillation under anesthesia as previously described.^18^

### Cell culture and treatments

A549 (human alveolar epithelial cell) and MRC-5 (human embryonic lung fibroblast) cell lines were purchased from the Cell Bank of the Chinese Academy of Sciences. Cells were maintained in Dulbecco’s modified Eagle’s medium and advanced minimum essential medium, respectively, and supplemented with 10% newborn calf serum, 100 U ml^−1^ penicillin, and 100 μg ml^−1^ streptomycin at 37 °C in a humidified atmosphere of 5% CO_2_ and 95% air. A549 or MRC-5 cells were treated with 5 ng ml^−1^ transforming growth factor beta 1 (TGF-β1) for 72 h.

### Hematoxylin and eosin and Masson’s trichrome staining

Pulmonary tissues were fixed by inflation with 4% paraformaldehyde overnight, dehydrated in 70% ethanol and embedded in paraffin wax. Sections of 4 mm thickness were prepared and subjected to hematoxylin and eosin or Masson’s trichrome staining as previously described.^16^

### Dual luciferase assays

The 3′UTR of the *ADAM* metallopeptidase domain 17 (*ADAM17*) was amplified using matched primers. The polymerase chain reaction (PCR) product was inserted into the vector. The accuracy of the construct was verified via direct sequencing. The seed sequence was then mutated by introducing a complementary sequence using primers. For the luciferase assay, 1 × 10^5^ 3T3 cells were harvested at 24 h after transfection and analyzed using a dual luciferase assay according to the manufacturer’s instructions. The firefly luciferase results were converted to a Renilla signal. All assays were repeated at least three times.

### Oligonucleotides and transfections

The *miRNA-708-3p* mimic, inhibitor and agomir were synthesized by RiboBio Co. Ltd. (Guangzhou, China). The *ADAM17* small interfering RNA was synthesized and purchased from GenePharma (Shanghai, China). All transfections were performed using the DharmaFECT1 reagent (Dharmacon, Austin, TX, USA) according to the manufacturer’s instructions.

### Transmission electron microscopy observation

Cells or lung tissues were fixed by treatment with fresh 3% glutaraldehyde at 4 °C for at least 4 h, post-fixed in 1% osmium tetroxide for 1.5 h, dehydrated in gradient ethanol, infiltrated with Epon812, embedded, and incubated at 37, 45 and 60 °C for 24 h. Ultrathin sections prepared with an ultracut E ultramicrotome (Leica, Wetzlar, Germany) were stained with uranyl acetate and lead citrate and observed using a JEM-1400 transmission electron microscopy system from Jeol Ltd. (Tokyo, Japan).

### Quantitative real-time PCR (qRT-PCR)

Total RNA was extracted using TRIzol reagent from Invitrogen (Carlsbad, CA, USA) as per the manufacturer’s instructions. Complementary DNA synthesis was performed using an M-MLV Reverse Transcriptase kit from Promega (Madison, WI, USA) following the manufacturer’s instructions. The qRT-PCR was performed using a SYBR Green PCR Master Mix kit from Takara Bio (Shiga, Japan) on the Rotor Gene3000 real-time PCR system from Corbett Research (Sydney, Australia).

### Western blot analysis

Total protein lysate samples containing 20 mg protein were subjected to 10% sodium dodecyl sulfate polyacrylamide gel electrophoresis, transferred onto polyvinylidene difluoride membranes, and blocked with 7% non-fat milk in Tris-buffered saline and Tween-20 (TBST; 50 mM Tris-HCl (pH 7.6), 150 mM NaCl, 0.1% Tween-20) for 1.5 h at room temperature. The membranes were washed three times with TBST buffer and incubated at 4 °C overnight with specific antibodies. After washing with TBST, the membranes were incubated with horseradish peroxidase-labeled IgG for 1.5 h at room temperature. The membranes were then washed with TBST, incubated with ECL reagent and exposed. The membranes were subsequently stripped and re-probed with a glyceraldehyde 3-phosphate dehydrogenase antibody, which served as a loading control. Each sample in each group was measured in triplicate, and the experiments were repeated at least three times.

### Analysis of promoter activity

Human genomic DNA was extracted from A549 cells by rapid isolation of mammalian DNA. The primers PF (5′-TTTCTCTATCGATAGGTACCGGCAGTTACCATTTCACAC-3′) and PR (5′-GATCGCAGATCTCGAGTAAAATGTGCCTACAAATA-3′) contain ends homologous to the pGL3-basic vector linearized with *Kpn*1 and *Xho*1. These primers were employed to amplify the 5′ promoter sequence of the *has-miR-708* gene from the extracted human genomic DNA. PCR was conducted at 94 °C for 5 min followed by 35 cycles at 94 °C for 45 s, 60 °C for 30 s and 72 °C for 2 min. The PCR-amplified fragment was approximately 1.1 kb in size and subcloned into the vector pGL3-basic by using the In-Fusion HD cloning kit from Clontech (Mountain View, CA, USA) Full-length reporter plasmids were transfected into A549 cells treated or not treated with TGF-β1.

### Statistical analysis

Statistical analysis was performed with the SPSS 17 software. All data were generated from a minimum of three independent experiments and expressed as the mean±s.d. Different groups were compared using Student’s *t*-test (for two groups) or one-way ANOVA (for more than two groups). *P*<0.05 was considered statistically significant.

## Results

### Decreased expression and upstream mechanism of *miR-708-3p* in IPF

**Figure 1:**
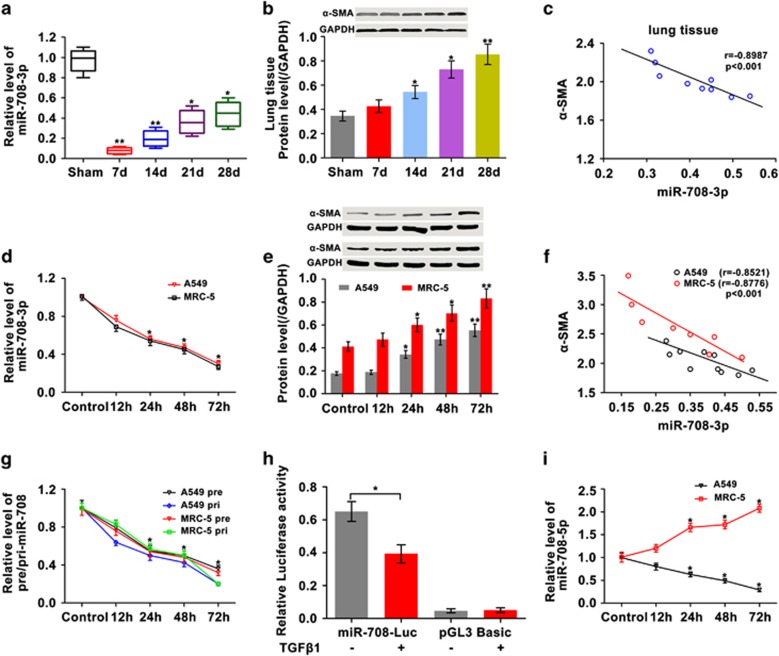
*MiR-708-3p* expression decreased during fibrosis *in vivo* and *in vitro*. (**a**) *MiR-708-3p* expression diminished in the lung tissues treated with BLM for 0, 7, 14, 21 and 28 days, as indicated by qRT-PCR. The *miR-708-3p* expression was normalized to the sham group. (**b**) α-SMA expression increased in the BLM group relative to that in the sham group. (**c**) Pearson correlation coefficient clarified that *miR-708-3p* inversely correlated with α-SMA *in vivo*. (**d**) The qRT-PCR results showed that *miR-708-3p* expression decreased in the TGF-β1-treated A549 and MRC-5 cells. (**e**) α-SMA increased in the TGF-β1-treated A549 and MRC-5 cells. (**f**) *MiR-708-3p* expression levels were inversely correlated with α-SMA *in vitro*. (**g**) The qRT-PCR results showed that *pri-miR-708* and *pre-miR-708* expression decreased in the TGF-β1-treated A549 and MRC-5 cells. (**h**) *MiR-708* promoter activity decreased in A549 cells treated with TGF-β1 for 72 h. The pGL3-basic indicates the negative control. (**i**) *MiR-708-5p* expression decreased in A549 cells and increased in MRC-5 cells treated with TGF-β1. Each bar represents the mean±s.d.; *n*=6. *P<0.05, **P<0.01.

In a previous study, we found that *miR-708-3p* exhibits more significant differences at low expression levels than those of other miRNAs in IPF.^16^ To verify our previous microarray analysis, *miR-708-3p* expression was evaluated in BLM-treated mice by qRT-PCR. The results showed that *miR-708-3p* expression decreased during fibrosis (Figure 1a). A physiological indicator of fibrogenesis, α-Smooth muscle actin (α-SMA), was tested. Western blot analysis showed that α-SMA was significantly more highly expressed in the BLM-treated group than in the sham group (Figure 1b). The Pearson correlation coefficient clarified that *miR-708-3p* inversely correlated with α-SMA (Figure 1c) and suggested that *miR-708-3p* expression may be inversely correlated with fibrosis.

Given the limitations of *miR-708-3p* transfection into animals, TGF-β1, the most important regulatory factor among the fibrogenic cytokines,^19, 20^ was used to stimulate human alveolar epithelial (A549) cells and human lung fibroblast (MRC-5) cells for confirmation of the *in vivo* results.^21, 22, 23^ The results showed that the *miR-708-3p* levels decreased and the α-SMA levels increased with the continued treatment of A549 and MRC-5 cells with TGF-β1 (Figures 1d and e). This result indicated that *miR-708-3p* was also inversely correlated with α-SMA *in vitro* (Figure 1f). These data supported the *in vivo* results and suggested that *miR-708-3p* may be regulated and may offer a novel target gene for managing IPF. Therefore, the regulatory mechanism of *miR-708-3p* should be further explored.

To investigate why the *miR-708-3p* levels decreased during fibrosis, we further studied the gene’s upstream regulators. First, *pri-miR-708* and *pre-miR-708* were assessed by qRT-PCR and were also found to be decreased in level (Figure 1g). The *miR-708*’s promoter activity was then tested and shown to be diminished (Figure 1h). *MiR-708-5p* is another miRNA derived from *pri-miR-708*; thus, *miR-708-5p* expression was analyzed. Interestingly, miR-708-5p decreased in the A549 cells and increased in the MRC-5 cells. Considering the cellular diversity and complexity of lung tissue, *miR-708-5p* was not further explored in this study. We inferred that *miR-708-3p* can be regulated by its upstream regulators.

### Function of *miR-708-3p* in IPF

**Figure 2:**
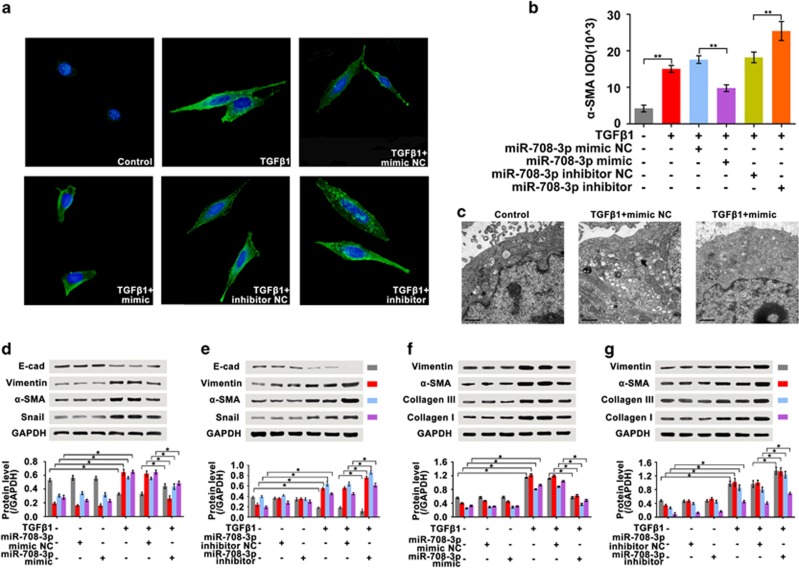
*In vitro* effect of *miR-708-3p* in IPF. A549 and MRC-5 cells were first transfected with *miR-708-3p* mimic or inhibitor for 6 h and then co-treated with TGF-β1 for 72 h. (**a**, **b**) Immunofluorescence analysis showed that the *miR-708-3p* mimic improved A549 cell shape and reduced α-SMA expression, but the *miR-708-3p* inhibitor induced the opposite results. (**c**) The *miR-708-3p* mimic improved the A549 ultrastructural changes, as observed by TEM. (**d**) The *miR-708-3p* mimic markedly promoted E-cadherin expression and diminished α-SMA, vimentin and Snail in A549 cells. (**e**) The *miR-708-3p* inhibitor markedly reduced E-cadherin and promoted α-SMA, vimentin and Snail expression in A549 cells. (**f**) The *miR-708-3p* mimic markedly decreased vimentin, α-SMA and collagen I and III expression in MRC-5 cells. (**g**) The *miR-708-3p* inhibitor markedly promoted vimentin, α-SMA and collagen I and III expression in MRC-5 cells. Each bar represents the mean±s.d.; *n*=6, *P<0.05, **P<0.01. TEM, transmission electron microscopy.

The *miR-708-3p* mimic and inhibitor were synthesized and transfected into A549 and MRC-5 cells treated with TGF-β1 for 72 h, after which the function of *miR-708-3p* was determined. During fibrosis development, epithelial cells acquire mesenchymal characteristics, including changes in cell shape, expression of mesenchymal markers, such as α-SMA, vimentin and transcription repressor Snail and loss of the epithelial cell marker epithelial cadherin (E-cadherin).^24^ Immunofluorescence analysis revealed that the treatment with TGF-β1 for 72 h altered the cell shape from irregular to spindle-like and that the α-SMA expression increased. Weak expression was associated with the group treated with the *miR-708-3p* mimic, whereas strong expression was noted in the group treated with the *miR-708-3p* inhibitor (Figures 2a and b). Cell ultrastructure improved and reverted to a near-normal state in the *miR-708-3p* mimic group (Figure 2c). A western blot showed that *miR-708-3p* mimic treatment markedly inhibited α-SMA, vimentin and Snail expression and promoted E-cadherin expression (Figure 2d). However, the *miR-708-3p* inhibitor yielded the converse of such effects relative to those of the TGF-β1-treated group (Figure 2e). To verify the A549 data, we analyzed these physiological indicators of fibrogenesis in the MRC-5 cells. The findings corroborated the A549 results (Figures 2f and g).

### Identification of the *miR-708-3p* target gene

**Figure 3:**
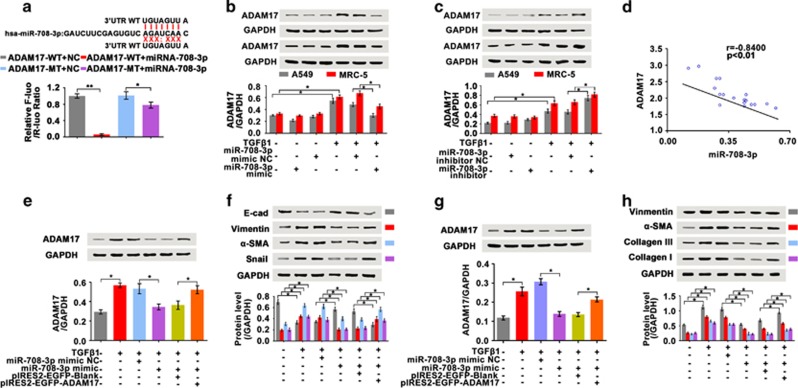
Identification of the *miR-708-3p*-targeted gene. (**a**) Sequences of *miR-708-3p* and the potential *miR-708-3p* binding site at the 3′UTR of *ADAM17*. The nucleotides mutated in the *ADAM17*-3′UTR mutant are also shown. The luciferase activity of WT *3′UTR-ADAM17* significantly decreased in the cells transfected with *miR-708-3p* mimics, whereas the *miR-708-3p* mimics could not inhibit the luciferase activity of MU *3′UTR-ADAM17*. (**b**) The *miR-708-3p* mimic inhibited *ADAM17* expression in A549 and MRC-5 cells. (**c**) The *miR-708-3p* inhibitor promoted *ADAM17* expression in A549 and MRC-5 cells. (**d**) Pearson correlation coefficient showed that *miR-708-3p* inversely correlated with *ADAM17*. (**e**–**h**) Rescue experiments (*miR-708-3p* mimic+*ADAM17* overexpression) confirmed *ADAM17* as the target of *miR-708-3p* in A549 and MRC-5 cells, respectively. Each bar represents the mean±s.d.; *n*=6, *P< 0.05, **P<0.01.

miRNAs exert regulatory functions by specifically interacting with their target genes. We first selected the predicted targeted genes of *miR-708-3p* on the basis of TargetScan, miRanda and miRbase data and analyzed their affinities. From the list of target genes of *miR-708-3p*, we focused on *ADAM17* because of its relatively high affinity. To verify whether *ADAM17* was a target gene of *miR-708-3p*, we constructed a luciferase assay reporter system by amplifying and inserting the 3′UTR of *ADAM17* into a vector containing the firefly luciferase downstream. The luciferase activity of WT *3′UTR-ADAM17* significantly decreased in the cells transfected with the *miR-708-3p* mimics, whereas the *miR-708-3p* mimics could not inhibit the luciferase activities of the MU *3′UTR–ADAM17*. These results suggest that *ADAM17* is the target gene of *miR-708-3p* (Figure 3a).

To further confirm *ADAM17* as a *miR-708-3p* target gene, we transfected the *miR-708-3p* mimic and inhibitor into A549 cells. *ADAM17* expression was inhibited by the *miR-708-3p* mimic but promoted by the *miR-708-3p* inhibitor (Figures 3b and c). Notably, the *ADAM17* level was inversely correlated with *miR-708-3p* expression (Figure 3d). Rescue experiments (*miR-708-3p* mimic+*ADAM17* overexpression) demonstrated that *miR-708-3p* directly regulated *ADAM17* expression (Figures 3e and f). To confirm the A549 data, we analyzed these indicators in the MRC-5 cells. The findings corroborated the A549 results (Figures 3g and h), which implies that *ADAM17* is the direct target gene of *miR-708-3p*.

### Regulation of *miR-708-3p* in IPF-associated aberrant signaling pathways

**Figure 4:**
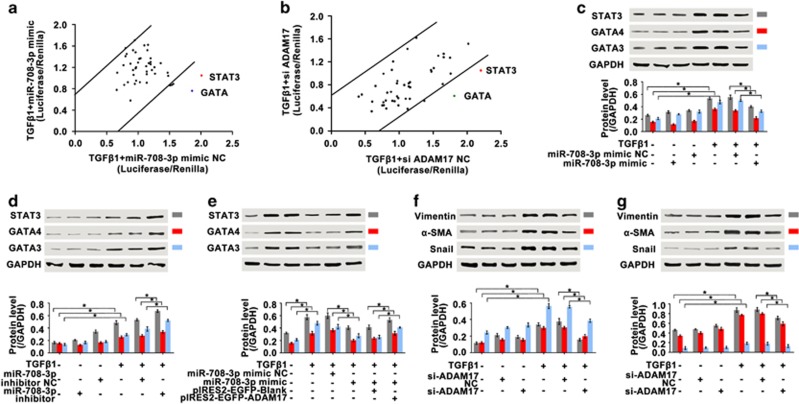
Regulation of *miR-708-3p* in the IPF-associated aberrant signaling pathways. (**a**) Identification of signaling pathways affected by the *miR-708-3p* mimic in MRC-5 cells. The *x* and *y* axes represent the normalized ratio of firefly/Renilla luciferase activities, respectively. Most of the significant changes were observed in the *STAT3* and *GATA* signaling pathways. (**b**) Identification of signaling pathways affected by *siADAM17* in MRC-5 cells. The *x* and *y* axes represent the normalized ratio of firefly/Renilla luciferase activities, respectively. Most of the significant changes were observed in the *STAT3* and *GATA* signaling pathways. (**c**) The *miR-708-3p* mimic inhibited the expression levels of *GATA3*, *GATA4* and *STAT3* proteins in A549 cells. (**d**) The *miR-708-3p* inhibitor promoted the expression levels of *GATA3*, *GATA4* and *STAT3* proteins in A549 cells. (**e**) Rescue experiments (*miR-708-3p* mimic+*ADAM17* overexpression) further confirmed *miR-708-3p* regulation in the IPF-associated aberrant *STAT3/GATA* signaling pathways in MRC-5 cells. (**f**) Si*ADAM17* blocked the expression of *GATA3*, *GATA4* and *STAT3* in A549 cells. (**g**) Si*ADAM17* blocked the α-SMA, vimentin and Snail expression in MRC-5 cells. Each bar represents the mean±s.d.; *n*=6, *P<0.05.

To further elucidate the mechanism by which the *miR-708-3p* axis regulates IPF, we evaluated the gene’s downstream signaling pathway. MRC-5 cells were transfected with the *miR-708-3p* mimic or *siADAM17* for 6 h and then co-treated with TGF-β1 for 72 h. Cignal Finder 45-Pathway analyzed the effects of the *miR-708-3p* mimic and *siADAM17* on the activation of key signaling pathways in human lung cells.^25^ In the reporter array, the *miR-708-3p* mimic significantly inhibited the *GATA/STAT3* reporter luciferase activities more effectively than the activities of the other reporters. Additionally, *siADAM17* significantly inhibited *GATA/STAT3* activity (Figures 4a and b).

The above-mentioned results suggest that *miR-708-3p*-induced aberrant fibrosis affects the *GATA/STAT3* signaling pathways through its target gene *ADAM17*. Thus, the *miR-708-3p* mimic and inhibitor, *siADAM17*, and the *ADAM17* overexpression vector were transfected into MRC-5 cells to confirm this point. Western blot analysis showed that the *miR-708-3p* mimic suppressed the expression of *GATA3*, *GATA4* and *STAT3* proteins, whereas the *miR-708-3p* inhibitor induced the opposite results (Figures 4c and d). Rescue experiments (*miR-708-3p* mimic+*ADAM17* overexpression) further proved the potential role of *miR-708-3p* in mediating the GATA/*STAT3*-dependent signaling pathway (Figure 4e). The effects of *ADAM17* on the pathway in human lung cells were tested using RNA interference technology (*siADAM17* and *ADAM17* overexpression). The results showed that *siADAM17* blocked the expression of *GATA3*, *GATA4* and *STAT3* (Figure 4f). In terms of fibrosis, *siADAM17* blocked α-SMA, vimentin and Snail expression (Figure 4g). All of these findings suggest that *miR-708-3p* induces aberrant fibrosis via the *GATA/STAT3*-dependent *ADAM17* signaling pathways.

### *MiR-708-3p* as potential therapeutic target for IPF

**Figure 5:**
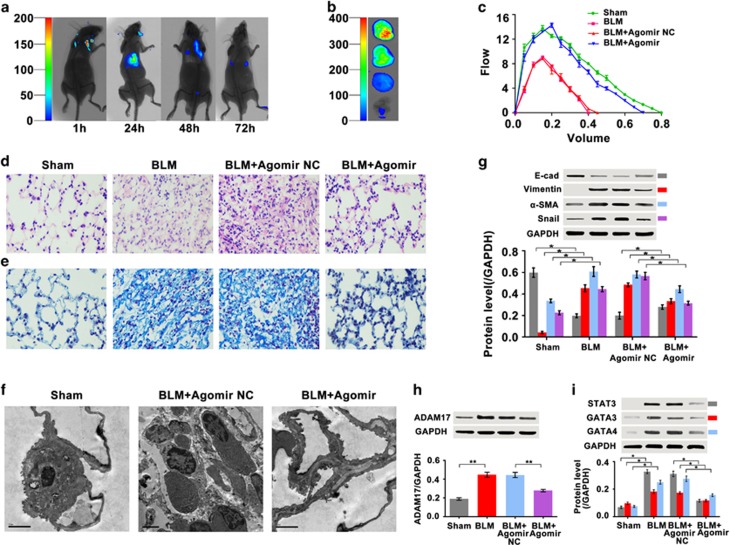
Anti-pulmonary fibrosis activity of *miR-708-3p* *in vivo*. (**a**) The *miR-708-3p* agomir, fluorescently labeled with Cy5, was tracked and observed to concentrate in the lung, as revealed by small-animal imaging technology. (**b**) The animal lungs were imaged at each time point through small-animal imaging technology. (**c**) The FVC% in the *miR-708-3p* agomir and sham groups was higher than those in the BLM group. (**d**) H&E staining revealed thinner alveolar walls of tissue sections and more continuous structures in the *miR-708-3p* agomir-treated group than in the BLM group (× 400 magnification). (**e**) Masson’s trichrome staining showed that *miR-708-3p* agomir treatment reduced the number of fibroblasts and the amount of collagen matrix relative to those in the BLM group. Blue stain denotes collagen (× 400 magnification). (**f**) Ultrastructural changes were observed in the cells of the *miR-708-3p* agomir and sham groups, as observed by TEM. (**g**) *MiR-708-3p* agomir promoted E-cadherin expression and inhibited α-SMA, vimentin and Snail expression. (**h**) *MiR-708-3p* agomir blocked *ADAM17* expression. (**i**) *MiR-708-3p* agomir diminished the *STAT3*, *GATA3* and *GATA4* expression levels. Each bar represents mean±s.d.; *n*=6. *P<0.05, **P<0.01. H&E, hematoxylin and eosin.

To evaluate the potential of *miR-708-3p* as a therapeutic target, we synthesized the *miR-708-3p* agomir for animal experiments. First, the *miR-708-3p* agomir was tracked *in vivo* using fluorescently labeled (Cy5) *miR-708-3p* agomir. By small-animal imaging technology, we assessed the drug delivery efficacy to the lungs, and the results showed that the dynamic image distribution of the *miR-708-3p* agomir mainly concentrated in the lungs *in vivo* (Figures 5a and b). The *miR-708-3p* agomir group showed a larger force vital capacity (FVC) than that of the BLM group (Figure 5c).

Thinner alveolar walls were observed in the tissue sections from the *miR-708-3p*-agomir-treated groups than from the BLM group. Masson’s trichrome staining showed that the number of fibroblasts and the amount of collagen matrix increased significantly in the BLM group, along with the formation of fibrotic lesions with a cordlike distribution. The *miR-708-3p* agomir treatment significantly reduced BLM-induced alveolitis and pulmonary fibrosis (Figures 5d and e). The cell ultrastructure improved and reverted to a near-normal state in the *miR-708-3p* agomir group, as observed in the transmission electron microscopy (Figure 5f). Western blot analysis was conducted to quantify the expression levels of E-cadherin, α-SMA, vimentin and Snail. The expression levels of α-SMA, vimentin and Snail were significantly higher, whereas those of E-cadherin were significantly lower, in the BLM group than in the sham group. *MiR-708-3p* agomir treatment markedly augmented E-cadherin expression and diminished α-SMA, vimentin and Snail expression (Figure 5g). Western blot analysis was also used to determine the effect of the *miR-708-3p* agomir on the targeted *ADAM17*, *STAT3* and *GATA* pathways. The results showed that *miR-708-3p* agomir blocked *ADAM17*, *STAT3*, *GATA3* and *GATA4* expression (Figures 5h and i). In general, *miR-708-3p* agomir treatment improved the histological and molecular markers of pulmonary fibrosis. All of these results were similar to those of the cell experiments. These data indicate that *miR-708-3p* is a potential therapeutic target for IPF.

### Clinical significance of *miR-708-3p* in patients with IPF

*MiR-708-3p* is highly conserved across species, but its expression in humans remains unexplored. Moreover, studies have not addressed whether the difference in expression levels was clinically significant between the patients with IPF and normal individuals. Therefore, we assessed the significance of the *miR-708-3p* correlation with IPF patients as claimed by the clinical research. Table 1 shows the characteristics and physiologies of patients with IPF and normal individuals. The number, age and gender of the normal individuals matched those of the patients with IPF. No statistical significance was observed among these characteristics. PaCO_2_ also showed no significant difference. By contrast, significant differences were noted in the percentages of predicted FVC (FVC% of predicted), total lung capacity (TLC) (TLC% of predicted), diffusion capacity for carbon monoxide (DLco% of predicted) and PaO_2_; these data are considered as physiological indicators of fibrogenesis during IPF progression. Hence, the indicators FVC% and DLco% were used to analyze the correlation of *miR-708-3p* expression with IPF.

**Figure 6:**
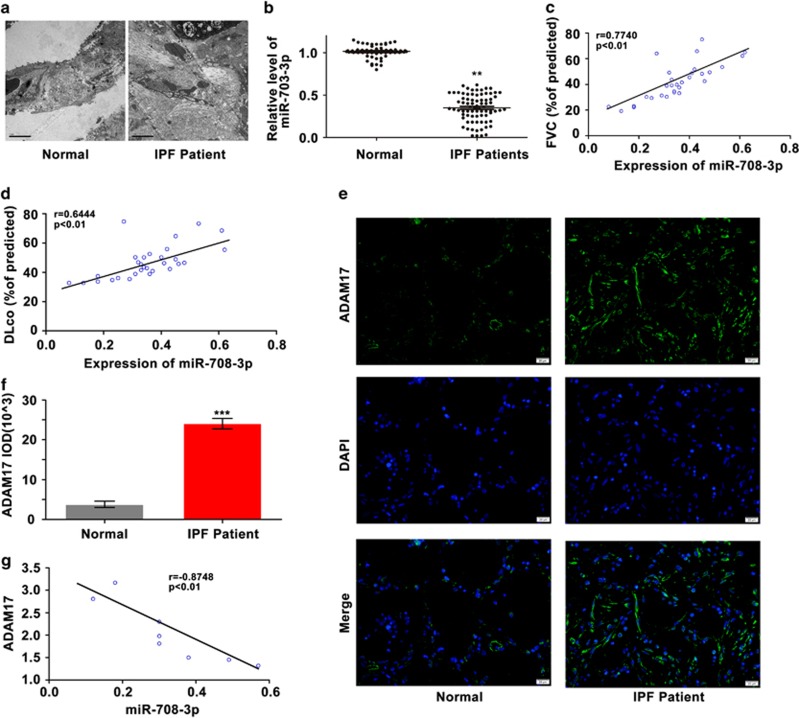
Clinical research of *miR-708-3p* in patients with IPF. (**a**) TEM showed an increased collagen amount in the patients with IPF relative to that in the normal subjects. (**b**) qRT-PCR confirmed that *miR-708-3p* expression was lower in the peripheral blood mononuclear cells of the patients with IPF than in those of the normal individuals. (**c**, **d**) *MiR-708-3p* levels positively correlated with FVC% and DLco%. Statistical analysis was performed using the Pearson correlation coefficient. (**e**, **f**) Immunofluorescence revealed that *ADAM17* expression increased significantly in the patients with IPF compared with normal individuals. (**g**) Pearson correlation coefficient showed that *miR-708-3p* inversely correlated with *ADAM17* in patients with IPF. Each bar represents mean±s.d.; *n*=6. ***P<0.01 compared with the normal group.

Transmission electron microscopy revealed low collagen expression in the normal subjects but high expression in the patients with IPF (Figure 6a). qRT-PCR was conducted to examine the *miR-708-3p* expression in peripheral blood mononuclear cells from patients with IPF. *MiR-708-3p* expression significantly decreased in the patients with IPF relative to that of normal individuals (Figure 6b). Pearson correlation coefficient analysis indicated that the *miR-708-3p* expression level positively correlated with the clinical pathological features, such as FVC% and DLco% (Figures 6c and d). The expression of the target gene *ADAM17* was detected by an immunofluorescence method. The results showed that *ADAM17* expression was significantly higher in the patients with IPF than in the normal population (Figures 6e and f). Meanwhile, the Pearson correlation coefficient showed that *miR-708-3p* inversely correlated with *ADAM17* in the patients with IPF (Figure 6g). These results coincided with those in our cell and animal IPF models and hence reflected the successful establishment of our models and the reliability of our *in vivo* and *in vitro* findings. These clinical results imply that regulating *miR-708-3p* expression may offer a novel biomarker or therapeutic approach for ameliorating IPF.

## Discussion

IPF is characterized by progressive fibrosis with a mortality of 50% in 3–5 years after diagnosis.^26^ High-resolution computed tomography (HRCT) is often used to identify patients with IPF at high risk of early death, but this diagnostic approach is considered unreliable.^27^ Molecular analysis for diagnostic purposes may enhance our understanding of the biological processes behind lung fibrosis, and its results may, in turn, aid IPF diagnosis.^28, 29^ A sensitive and specific tool for early diagnosis may be the detection of predictive markers in blood samples by liquid biopsy.^30^ However, only a few studies have reported on blood markers in IPF, especially for miRNA. In the present study, we discovered that *miR-708-3p* is downregulated in patients with IPF and that *miR-708-3p* can target *ADAM17* through the *GATA/STAT3* signal pathway to control IPF initiation and progression. Our findings have provided a potential diagnostic marker or therapeutic target for IPF.

MiRNAs implicated in diseases represent a promising class of therapeutic targets. Researchers have long been interested in targeting miRNAs by using oligonucleotides (miRNA inhibitors or mimics) as a strategy.^31, 32^ However, only a few studies have documented the therapeutic targeting of miRNA in IPF. *MiR-708* is processed from the first intron of the teneurin transmembrane protein 4 gene located on chromosome 11. The mature sequence of *hsa*-*miR-708-3p* is designated with the accession number of MIMAT0004927; its previous ID was hsa-miR-708*, and the sequence was 57 – caacuagacugugagcuucuag – 78. Numerous studies on *miR-708* exist, whereas few works have explored *miR-708-3p*/miR-708*.^33, 34, 35, 36^ Recently, *miR-708* has been identified to be transcriptionally repressed by polycomb-repressor-complex-2-induced H3K27 trimethylation in metastatic breast cancer.^37^ However, to date, no study has detailed the mechanism of *miR-708-3p*. *MiR-708-3p* is downregulated in prostate carcinoma and pleuropulmonary blastoma,^38, 39^ but its regulatory mechanism remains unexplored. In the present study, we systematically and rigorously investigated the role and regulatory mechanism of *miR-708-3p* in IPF. We were unable to use more profiles than those adopted in our study for *miR-708-3p* in the IPF tissue because of the lack of lung parenchyma samples. However, because the miRNAs detected at altered levels in serum^40, 41, 42, 43^ are often dysregulated in malignant tissues,^44^ serum profiling may be used directly to identify candidate biomarkers.^40, 45^ Therefore, our study provides a rationale for developing *miR-708-3p* as a biomarker or therapeutic agent for IPF.

The current study focused on the target gene *ADAM17*, which acts as a molecular switch for controlling immune responses, tissue regeneration and cancer development.^46^ *ADAM17* is expressed and upregulated in tumor cells almost ubiquitously^47^ and is rarely reported in fibrotic diseases. However, *ADAM17* has been detected in cleavage-activated proligand substrates, particularly for the epidermal growth factor receptor (EGFR) ligand amphiregulin in kidney fibrosis.^48^ As a consequence, EGFR is persistently activated and triggers the synthesis and release of proinflammatory and profibrotic factors, thereby resulting in fibrosis. Targeting the *ADAM17* pathway presents a therapeutic target for fibrosis or cancer.^46, 47, 48, 49, 50^ However, reports have not indicated the involvement of *ADAM17* upstream regulators, such as miRNA, in the regulation of the *ADAM17* pathway. In this paper, we were the first to report *ADAM17* as a target gene of *miR-708-3p*, which can regulate *ADAM17* by base pairing with the binding site in the 3′UTR of the protein to control fibrogenesis. Therefore, fibrogenesis is affected by interference with *miR-708-3p* or *ADAM17*. miRNA is considered more sensitive and shorter than mRNA and has its own unique advantages.

Our results strongly indicate the interplay of the *GATA/STAT3* signaling pathways, the physiological *ADAM17* downstream pathways. *GATA* is zinc-finger transcription factor linked to cell differentiation. *GATA6* participates in the differentiation of fibroblasts into myofibroblasts by mediating the α-SMA-inducing signal of TGF-β1 in IPF.^51^ Moreover, the overexpression of *GATA3* enhances the development of pulmonary fibrosis by reducing interferon levels in the lungs.^52^ *STAT3* activation is abundant in the fibrotic lungs of patients with IPF, and the genetic reduction of *STAT3* protects the mice from BLM-induced lung fibrosis.^53^ *STAT3* contributes to pulmonary fibrosis through epithelial injury and fibroblast–myofibroblast differentiation.^54, 55^ We analyzed the causes of significant abnormality in the *GATA* and *STAT3* signaling pathways. We speculated that although IPF is characterized by excessive collagen deposition and subsequent fibrosis, inflammation cannot be ignored during IPF development. The two pathways are closely related to inflammatory cytokines. Therefore, aberrant *miR-708-3p* expression induced significantly changed abnormalities in the *GATA* and *STAT3* pathways.

Finally, we assessed the significance of the correlation of *miR-708-3p* with IPF, as reported by clinical research, and demonstrated the best clinical evidence for patients with IPF. Our *in vivo* and *in vitro* findings, as well as the clinical data from patients with IPF, supported the regulatory mechanism of *miR-708-3p* in IPF progression. Furthermore, the therapeutic potential of targeting IPF-downregulated miRNA by oligonucleotides showed the pharmacological value of miRNA in IPF. Future studies are required to expand the analysis of *miR-708-3p* to a larger cohort of patients with IPF to determine with statistical confidence whether reduced *miR-708-3p* expression correlates with a worsened patient outcome.

## Figures and Tables

**Table 1:** Characteristics and physiologies of IPF patients and normal individuals

*Characteristic*	*Normal*	*IPF*	*P-value*
Number	78	78	/
Age (years)	67.9±7.5	68.1±6.8	/
Gender (Male/female)	59/19	54/24	/
FVC (% of predicted)	88.7±10.7	57.8±9.3	<0.01
TLC (% of predicted)	90.7±9.6	63.6±10.9	<0.01
DLCO (% of predicted)	85.8±5.3	57.2±4.9	<0.01
PaO2 (mm Hg)	89.7±4.6	67.2±5.9	<0.01
PaCO2 (mm Hg)	38.2±4.7	35.2±3.7	/
Smoking History (*n*)	49	51	/

Abbreviation: DLCO, diffusing capacity for carbon monoxide; FVC, forced vital capacity; IPF, idiopathic pulmonary fibrosis; TLC, total lung capacity; smoking history denotes subjects with >5 years of cigarette smoking.
